# Measurement of Polycyclic Aromatic Hydrocarbons in Baby Food Samples in Tehran, Iran With Magnetic-Solid-Phase-Extraction and Gas-Chromatography/Mass-Spectrometry Method: A Health Risk Assessment

**DOI:** 10.3389/fnut.2022.833158

**Published:** 2022-02-17

**Authors:** Mojtaba Moazzen, Nabi Shariatifar, Majid Arabameri, Hedayat Hosseini, Mahsa Ahmadloo

**Affiliations:** ^1^Department of Food Technology Research, Faculty of Nutrition Sciences and Food Technology/National Nutrition and Food Technology Research Institute, Shahid Beheshti University of Medical Sciences, Tehran, Iran; ^2^Department of Environmental Health Engineering, School of Public Health, Tehran University of Medical Sciences, Tehran, Iran; ^3^Food Safety Research Center (salt), School of Nutrition and Food Sciences, Semnan University of Medical Sciences, Semnan, Iran; ^4^Department of Food Science and Technology, National Nutrition and Food Technology Research Institute, Faculty of Nutrition Sciences and Food Technology, Shahid Beheshti University of Medical Sciences, Tehran, Iran; ^5^Department of Food Safety and Hygiene, School of Public Health, Qazvin University of Medical Sciences, Qazvin, Iran

**Keywords:** baby foods, GC/MS, health risk assessment, polycyclic aromatic hydrocarbons, MSPE

## Abstract

Baby food is one of the most sensitive foods available, which is closely monitored for carcinogens. In this study, 16 Polycyclic Aromatic Hydrocarbon (PAH) compounds were evaluated by using the method of magnetic-solid-phase-extraction and gas-chromatography/mass-spectrometry (MSPE/GC-MS). The recovery, limit of detection (LOD), and limit of quantification (LOQ) of PAH compounds were 93.4–101.6%, 0.06–1.12, and 0.18–3.38 μg/kg, respectively. The results indicated the mean of total PAHs in all samples was 3.73 ± 0.8 μg/kg, and the mean of Benzo[a]pyrene (BaP) was 0.29 ± 0.14 μg/kg that were lower than the USA-Environmental Protection Agency (USEPA) standard level (1 μg/kg, BaP in baby foods). In addition, our results showed that mixed five cereal-based baby food had a maximum mean of ΣPAHs (5.06 ± 0.68 μg/kg) and mixed wheat and date-based baby food had a minimum mean of ΣPAHs (3.03 ± 0.41 μg/kg). The carcinogenic risk due to PAH in the tested baby foods sold in Iran was adequately low, and all examined products were safe for consumers. Therefore, it can be said that the consumption of baby foods does not pose a threat to consumers.

## Introduction

Today, numerous carcinogenic and dangerous compounds are identified in the environment and food, one of them is Polycyclic Aromatic Hydrocarbon (PAH) compound, which may be detected in food, water, and air. High PAH levels in unprocessed foods in most areas may be due to volcanoes, forest fires, the oil industry and other industries, trucks, and so on. A positive association has been reported between PAH exposure and the incidence of complications, such as gastric cancer, lung problems, and cytogenetic and biochemical changes (1–4).

The USA-Environmental Protection (USEPA) has listed 16 of these compounds in the list of sustainable food contaminants. Benzo[a]pyrene (BaP) is categorized as category 2A (possibly carcinogenic) to humans by the International Agency for Research on Cancer (IARC). The European Union (EU) has set a limit of <1 μg/kg BaP for processed cereal-based processed foods for children and infants (5–8).

Polycyclic Aromatic Hydrocarbons have been shown to accumulate in a variety of food matrices and may enter the body in three routes: dermal, respiratory, and swallowing. During packaging, cooking, processing, and heat activities (such as grilling, smoking, frying, and cooking), PAHs are produced in a variety of foods (9–11). In addition, the transfer of PAH from the environment (air, soil, and water) to agricultural products (such as cereals) can be significant, especially when farms are located near industrial sites or factories (1, 12–15).

In recent years, cereal-based baby food has become a key part of a baby's daily diet. Regardless of cultural or religious concerns, baby meals and formulas have a high priority in strategies aiming at promoting good child health (16–20). Therefore, due to the weakness of the child's immune system, it is necessary for the food used by this group to be constantly examined and evaluated for contaminants in order to pose a lower risk to them (16, 17, 21, 22).

Various sample pretreatment procedures, such as solid phase microextraction (SPME), solid phase extraction (SPE), and stir bar sportive extraction (SBSE), have been applied for separation of PAH and preconcentration in baby food samples in recent years (17, 23, 24). One of the SPE procedure limitations is that adsorbents must be put in a difficult-to-use SPE cartridge. Manual operation and memory effects are further disadvantages of the SBSE procedure (13, 25–30). Adsorbents are divided from the aqueous phase by filtering or centrifuging in SPME that can take a long period when dealing with large sample volumes. Furthermore, in SPME, the coatings of polymer are brittle and fibers of SPME are relatively expensive. Magnetic-solid-phase-extraction (MSPE), which is based on magnetic nanoparticles, has lately been introduced as a viable approach for sample preparation. Adsorbents (magnetized) are disseminated directly and consistently in the solution of the sample, in the MSPE approach. In addition, the objective components (contaminate compounds) are adsorbed on the matrix of adsorbent and divided from the sample solution via a magnet (external) utilized outside the extraction vessel (7, 12, 14). As a result, PAH analytes are removed from adsorbents (magnetized) using an organic solvent for further examination. Lastly, the eluted extracts are examined by chromatography of liquid or gas (HPLC or GC) and various detectors, such as mass fluorescence (FID) or (MS) (1, 5, 14, 15).

As it turns out, health risk assessment is an important perspective of a risk management program (20, 31). Therefore, health risk assessments are performed to show different possible mixtures by estimating adverse effects on health exposure conditions (30, 32). The Monte Carlo method improves the knowledge of the environmental behavior of PAH in terms of their health hazards through variable distributions (33, 34).

Since very little research has been done on the presence of PAH compounds in baby foods in Iran and the world, the objectives of this study are (1) to develop magnetic-solid-phase-extraction and gas-chromatography/mass-spectrometry (MSPE-GC/MS) method to measure PAH compounds in baby foods, (2) to measure 16 PAH compounds in baby food samples (rice-based, wheat-based, mixed wheat and rice-based, mixed wheat and honey-based, mixed wheat and date-based, mixed almond porridge and fruit-based, mixed wheat and fruit-based, mixed five cereal-based, and almond porridge-based) of Iran, and (3) to estimate the potential health risk of PAHs in humans.

## Materials and Methods

### Chemical Compounds and Standards Preparation

Sigma Aldrich (Steinheim, Germany) provided PAHs certified reference material (QTMPAH-Mix, 2,000 μg/ml, CRM47930). The standards were produced in dichloromethane (100 μg/ml) that was then diluted with dichloromethane/methanol solution (50:50, v/v) for additional dilutions. Biphenyl (Merck Co., Kenilworth, NJ, USA) was made as an internal standard (I.S.) in methanol (0.05 μg/ml). The solutions of stock and working were kept at 4°C. From the Nanoshel Co. (Panchkula, India), the multi-walled carbon nanotubes modified with iron oxide and silver nanoparticles (MWCNTs) as adsorbent (diameter 30–60 nm, length 5.0–30 μm) were purchased. The MWCNTs-Fe_3_O_4_ (magnetized adsorbent) combination was ready based on the recent research (7, 15).

### Blank Sample Preparation

For the preparation of a blank sample, distilled water was chosen. By preliminary research studies, its suitability as the blank sample was confirmed (7, 15).

### Sampling

Thirty-six baby food samples [consist of (1) rice-based; (2) wheat-based; (3) mixed wheat and rice-based; (4) mixed wheat and honey-based; (5) mixed wheat and date-based; (6) mixed almond porridge and fruit-based; (7) mixed wheat and fruit-based; (8) mixed five cereal-based (wheat, rice, corn, barley, and oat); (9) almond porridge-based] were bought randomly (in duplicate) in Tehran, Iran, among two well-known brands (A and B). The samples were stored in their original packaging, properly labeled, and refrigerated (4 ± 1°C) until they were analyzed in the lab.

### Samples Preparation

The steps of samples preparation involve three key phases: sample clean-up, of the compounds adsorption, and the desorption of compounds from adsorbent, which are described in detail in our previous research (7, 15).

### Analytical and Instrumental Conditions

In this research, the model of gas chromatography device was Agilent (6890, Palo Alto, CA, USA) and the model of the detector of mass was Agilent (5973, Palo Alto, CA, USA). Other instrumental and analytical conditions were according to our previous research studies (7, 15). Selected ions, time window, confirmation ions, and quantification ions are presented in Table 1.

**Table 1:** Selected ions applied for the qualification and quantification of PAH compounds by GC-MS (SIM mode).

**Ion group**	**Analyte (PAHs)**	**Time window (min.)**	**Confirmation ions (m/z)**	**Quantification ion (m/z)**
1	I.S. (Biphenyl)	8–13	153, 152	154
1	NA (Naphthalene)	6–13	128, 127	128
1	Ace (Acenaphthylene)	8–13	153, 151	152
1	Ac (Acenaphthene)	8–13	154, 152	153
2	F (Fluorene)	13–15	165, 167	166
3	Pa (Phenanthrene)	15–17	179, 176	178
3	A (Anthracene)	15–17	179, 176	178
4	Fl (Fluoranthene)	17–20	203, 101	202
4	P (Pyrene)	17–20	203, 101	202
5	BaA (Benzo[a] anthracene)	20–23	226, 229	228
5	Ch (Chrysene)	20–23	226, 229	228
6	BbF (Benzo[b] fluoranthene)	23–28	253, 126	252
6	BkF (Benzo[k] fluoranthene)	23–28	253, 126	252
6	BaP (Benzo[a]pyrene)	23–28	253, 126	252
7	IP (Indeno[1,2,3-cd]pyrene)	28–31	277, 138	276
7	DhA (Dibenzo[a,h] anthracene)	28–31	279,139	278
7	BgP (Benzo[g,h,i] perylene)	28–31	277,138	276

*PAH, PAHs, Polycyclic Aromatic Hydrocarbons; GC/MS, gas-chromatography/mass-spectrometry*.

### Characterization of Human Health Risk

In order to estimate the oral exposure dose of the harmful compound, such as PAH, the daily ingestion and the incremental lifetime cancer risk (ILCR) index of indicator PAH via the ingestion of baby foods products were estimated by Equations (1) and (2) based on previously published methods (8, 35):


(1) $$\begin{array}{r} BEC=\overset{n}{\underset{i=1}{\sum }}C_{i}\times TEF \end{array}$$

(2) $$\begin{array}{r} EDI\;=\;\frac{C\;\times \;IR\;i\times \;EDi\;\times \;EFi}{BW\;\times \;AT} \end{array}$$

In this equation, estimated daily intake (EDI) is based on the mg/kg, C is the concentration of PAH based on mg/kg, and the definition and description of the above variables are shown in Table 2. PAH concentrations were converted to BaP equivalent concentrations (BEC; μg/kg) by toxicity equivalency factors (TEFs). The ILCR from exposure to PAH (BaP from Group 2A, a probable human carcinogen) through baby foods products is another approach to evaluate the risk and were calculated using Equation (3):


(3) $$\begin{array}{r} ILCR=\;\frac{BEC\times EF\times ED\times SF}{BW\;\times \;AT} \end{array}$$

where *SF* denotes the oral cancer slope factor of the BaP daily intake (7.3 per mg/kg/d) [USEPA (36), Washington, DC, USA]. Risk assessment guidance for Superfund: volume III part A and risk assessment.), and the definition and description of variables are shown in Table 2.

**Table 2:** Parameters used in the present study for health exposure assessment in baby foods.

	**Exposure parameters**	**Unit**	**Reference**
SF	Carcinogenic slope factor of oral intake (7.3)	[mg/(kg/d)]	(USEPA. Risk assessment guidance for Superfund: volume III part A and risk assessment. Washington) (36)
C	Concentrations of PAHs	μg/kg	–
EDI	Estimated daily intake	Mg/kg	
*EFi*	Exposure frequency	days per year	Karimi et al. (39)
IR	Average daily baby foods intake (means based on manufactory order)	kg/day	Kalantari (41), Badibostan et al. (18)
ED	Exposure duration	days	Roudbari et al. (7)
BEC	Benzo(a)pyrene equivalents concentrations by toxicity equivalency factors (TEFs)	–	EPA (42)
AT	Average time	days	Eghbaljoo-Gharehgheshlaghi et al. (43)
BW	Body weight (70)	kg	Shariatifar et al. (33)
TEFs	Toxic equivalent factors	–	Supplementary Table 1

### Statistical Analysis

The findings were statistically evaluated by SPSS V.24. The data were stated as mean ± SD. The *t*-test and independent one-way ANOVA were used to determine statistical significance. For comparing means of PAH level according to types of baby food, we employed a one-way ANOVA. When *p* < 0.05, values were considered statistically significant. In situations where the components of PAH could not be found, half of limit of detection (LOD) was used to determine the mean level. A heat map was conducted to ascertain a more accurate distinction between the PAH congener in baby foods (15, 37). Heat map construction (clustering method: average linkage; distance method: Pearson) was applied to interpret the association between individuals online at https://biit.cs.ut.ee/clustvis. The Crystal Ball software (V 11.1.2.4.600) was employed to produce simulation predictions (33).

## Results and Discussion

### Performance and the Analytical Method Validation

According to Table 3, the optimum conditions are supplied for study, and calibration curves (0.05–10 μg/kg) are drawn with a correlation coefficient ranging from 0.982 to 0.997. The limits of quantification (LOQs) and limits of detection (LODs) for the PAH compounds were changed to 0.18–3.38 and 0.06–1.12 μg/kg, respectively, according to the validation tests. The inter-day precision of the procedure was evaluated using analytical QC samples ready at four levels on 3 repeated days. The estimated values for repeatability and reproducibility were 6.7–10.7 and 7–18%, respectively. For testing the percent of recovery and accuracy of the procedure, we used PAHs certified reference material (QTMPAH-Mix, 2,000 g/ml, CRM47930) (5, 8, 14). The recovery rates were ranged from 93.4 to 101.6%. As a result, the reliability and feasibility of the developed procedure were proven. The method's selectivity was verified by testing 32 samples of baby food. Lastly, no interfering peaks were seen in the vicinity of the internal standard and analytes.

**Table 3:** Linear range, LOD, LOQ, *r*^2^, recovery, RSDr (*n* = 6), and RSDR (*n* = 6) of developed method.

**Target compound**	**Linear range (μg/kg)**	**Limit of detection (LOD) (μg/kg)**	**Limit of quantification (LOQ) (μg/kg)**	**Coefficient of estimation (r2)**	**Recoveries (%)**	**Repeatability (RSDr) (%)**	**Reproducibility (RSDR) (%)**
Na	0.05–10	0.08	0.25	0.988	100.4	10.7	9, 12, 17
Ace	0.05–10	0.34	1.02	0.992	93.4	6.7	11, 8, 15
Ac	0.05–10	0.26	0.78	0.982	97.7	8.2	10, 9,16
F	0.05–10	0.6	1.81	0.993	95.3	9.1	12, 14, 18
Pa	0.05–10	0.18	0.55	0.985	101.6	10.2	8, 12, 16
A	0.05–10	0.22	0.67	0.995	94.9	9.6	7, 10, 15
Fl	0.05–10	0.54	1.62	0.988	96.4	8.5	9, 12, 15
P	0.05–10	0.22	0.67	0.997	100.8	9.1	10, 14, 18
B(a)A	0.05–10	0.12	0.37	0.990	101.1	9.9	9, 10, 15
Ch	0.05–10	0.32	0.96	0.996	95.6	7.8	9, 12, 16
B(b)F	0.05–10	0.08	0.25	0.986	99.2	7.7	8, 9, 12
B(k)F	0.05–10	0.42	1.27	0.987	96.5	8.4	9, 11, 13
B(a)P	0.05–10	0.22	0.67	0.992	100.5	8.5	10, 14, 16
D(h)A	0.05–10	0.08	0.25	0.996	97.7	9.8	8, 10, 15
B(g)P	0.05–10	1.12	3.38	0.986	99.8	10.1	9, 11, 14
I(cd)P	0.05–10	0.06	0.18	0.983	96.6	8.6	9, 13, 16

*RSDR of 1, 5, and 10 μg/kg standard value (n = 6)*.

### Evaluation of PAH Compounds in Baby Foods Samples

The statistical analysis of the PAH compounds in bottled water is demonstrated in Table 4. The results showed that the mean level of total PAHs was 3.73 ± 0.8 μg/kg. The mean of BaP levels was 0.29 ± 0.14 μg/kg that ranged from non-detected (nd) to 0.58 μg/kg that was lower than the EU standard level (1 μg/kg of BaP), which can be due to the use of appropriate (in accordance with the standard of Iran) cereals and other additives, although the strict and continuous monitoring of related organizations (such as Iran Food and Drug Administration and Standard Organization of Iran) can also be another reason for the good quality of these products in terms of the amount of this contaminant. The mean of PAH4 was 0.72 ± 0.22 μg/kg that was lower than the regulation (EC) no. 1881/2006 (1 μg/kg for PAH 4 in processed cereal-based foods and baby foods for infants and young children) that lower concentration of PAH 4 can be a good sign of the high quality of baby food samples in Iran, which can be due to the high quality of raw ingredients used (cereals and other additives), the absence of secondary contaminants (such as contaminants of water, air, Factory equipment, and packaging materials). The maximum mean of PAHs was pyrene (P) (0.45 ± 0.15 μg/kg), and also, the minimum mean of PAHs was acenaphthene (Ac), fluoranthene (Fl), benzo[a] anthracene (BaA), and benzo[g,h,i] perylene (BgP) that was nd in all samples. The higher levels of some analytes (such as P) in samples can be due to certain contaminants, because no standard has been defined for them, so no specific comparison or explanation can be given.

**Table 4:** Statistical analysis of PAHs in baby food samples (μg/kg).

	**Min**	**Max**	**Mean**	**SD**
**NA**	Nd	0.3	0.16	0.08
**Ace**	Nd	0.45	0.20	0.08
**Ac**	Nd	nd	Nd	–
**F**	Nd	0.89	0.42	0.20
**Pa**	Nd	0.36	0.14	0.09
**A**	Nd	0.45	0.23	0.12
**Fl**	Nd	nd	Nd	–
**P**	0.28	0.72	0.45	0.15
**BaA**	Nd	nd	Nd	-
**Ch**	Nd	0.41	0.29	0.11
**BbF**	Nd	0.21	0.08	0.05
**BkF**	Nd	0.59	0.36	0.14
**BaP**	Nd	0.58	0.29	0.14
**DhA**	Nd	0.14	0.05	0.03
**IP**	Nd	0.21	0.05	0.05
**BgP**	Nd	nd	Nd	–
**Total**	2.7	5.54	3.73	0.80
**PAH4**	0.37	0.98	0.72	0.22

*PAHs, Polycyclic Aromatic Hydrocarbons; nd, non-detected*.

Domfeh in 2021 measured PAHs in baby foods based on cereal and reported that the mean concentrations of ΣPAHs (15 PAHs), ΣPAH4 [BaA, chrysene (Ch), BaP, and benzo[b] fluoranthene (BbF)], and BaP were 276.7 ± 256.2, 16.0 ± 32.2, and 5.0 ± 9.0 μg/kg, respectively (38). Badibostan et al. measured four PAHs in baby food samples and reported that the mean level of BaP was ranged from nd to 0.73 ± 0.06 μg/kg, which was somewhat similar to our research (18). Soceanu et al. measured 15 PAH compounds in baby food samples and reported that the mean level of total PAHs ranged from 2.52 ± 0.22 to 6.7 ± 0.375 μg/kg (that was higher than our outcomes) and the mean level of BaP was varied from <0.01 to 0.26 ± 0.00 μg/kg that was somewhat similar to our results (17). Cai et al. measured four PAHs in infant formula powders and reported that the PAH4 level was detected to be in the variety of 0.1–0.87 μg/kg (mean level 0.4 μg/kg) that was somewhat similar to our findings, and also, the mean level of BaP was 0.021 μg/kg (varied from <0.1 to 0.09 μg/kg) that was lower than these findings (19). Ciecierska et al. measured 19 PAHs in baby food samples and reported that the mean level of ΣPAHs was varied from 0.28 ± 0.04 to 7.45 ± 0.82 μg/kg (that were higher than our findings), and the mean level of BaP was ranged from nd to 0.25 ± 0.06 μg/kg that was similar to our results (21). Han et al. measured eight PAHs in infant formula samples (Korea) and reported the mean level of eight PAHs was 0.181 ± 0.003 μg/kg and the mean level of BaP was 0.056 ± 0.001 μg/kg that was less than this research (16). Iwegbue et al. measured 16 PAHs in infant formula in Nigeria and reported the levels of the 16 PAHs in infant formulae (for infants of ages 0–12 months, 1–3 years, 6–12 months, and 0–6 months) varied 0.51–0.70, 0.054–1.98, 0.081–2.54, and 0.102–1.98 μg/kg, respectively. In all samples, the BaP level was below the 1 μg/kg that was similar to our findings (24). Rey-Salgueiro et al. measured 11 PAHs in infant food samples and reported in only two samples, one cereal sample and one milk, B[k]F was identified at levels of 0.30 and 0.10 μg/kg, respectively (that were somewhat similar to our findings) and other PAH compounds were not detected (23). Santonicola et al. measured 14 PAHs in baby foods based on meat/fish in Italy and reported that the average of total PAHs was 11.82 μg/kg in samples (which was upper than this research), and the mean of BaP was 0.19 μg/kg that was somewhat similar to our results (22).

Higher or lower levels of PAH contaminants in baby food are mainly related to the level of contamination of their raw materials (cereals and other additives), although other reasons, such as secondary contamination transmitted from devices and packaging materials, can also be mentioned. Contamination of raw materials with PAHs can be from numerous sources like environmental contamination (water, soil, and air), proximity to industrial centers, proximity to municipal wastewater, proximity to highways, and other centers.

### Evaluation of PAH Compounds in Baby Food Samples

Statistical analyses of PAHs in baby foods (rice-based, wheat-based, mixed wheat and rice-based, mixed wheat and honey-based, mixed wheat and date-based, mixed almond porridge and fruit-based, mixed wheat and fruit-based, mixed five cereal-based, and almond porridge-based) are presented in Table 5. Between different types of baby food about PAH contaminants, there was no statistically significant difference. Our results showed that mixed five cereal-based baby food had a maximum mean of? PAHs (5.06 ± 0.68 μg/kg) and mixed wheat and date-based baby food had minimum mean of? PAHs (3.03 ± 0.41 μg/kg), the reason for the higher level of contamination in mixed five cereal-based baby food is probably due to the contamination of one or more of the cereals used, because usually in Iran, one or two common cereals are used, namely, wheat and rice, therefore, other cereals used may be less monitored, although was lower than the existing standards. The BaP level was lower than EU standard in all samples (lower than 1 μg/kg), the maximum mean of BaP was detected in mixed almond porridge and fruit-based baby foods (0.55 ± 0.04 μg/kg), and minimum mean of BaP was identified in rice-based and mixed wheat and honey-based baby food (nd), which can be more contaminated than almond porridge and fruit than other additives. The maximum mean of PAH4 was detected in mixed five cereal-based baby food (0.98 ± 0.02 μg/kg) and minimum mean of PAH4 was detected in rice-based baby food (0.47± 0.14 μg/kg) that in all samples was lower than the regulation (EC) no. 1881/2006 (1 μg/kg for PAH 4 in processed cereal-based foods and baby foods for infants and young children). Although the amount of PAH 4 is lower than the existing standards (EU), the reason for the higher contaminant in samples of the mixed five cereal-based baby food is probably due to the use of several types of cereals, such as corn, which is usually imported and has increased the possibility of contamination by mixing with other cereals.

**Table 5:** Statistical analysis of PAHs in baby food samples (μg/kg).

		**NA**	**Ace**	**Ac**	**F**	**Pa**	**A**	**Fl**	**P**	**BaA**	**Ch**	**BbF**	**BkF**	**BaP**	**DhA**	**IP**	**BgP**	**Total**	**PAH4**
**Rice-based**	Mean	0.18	nd	nd	nd	nd	0.37	Nd	0.47	nd	0.26	nd	nd	nd	nd	nd	nd	3.28	0.47
	SD	0.08	–	–	–	–	0.08	–	0.02	–	0.14	–	–	–	–	–	–	0.32	0.14
	Max	0.23	nd	nd	nd	nd	0.42	nd	0.48	nd	0.36	nd	nd	nd	nd	nd	nd	3.50	0.57
	Min	0.12	nd	nd	nd	nd	0.31	nd	0.45	nd	nd	nd	nd	nd	nd	nd	nd	3.05	0.37
**Wheat-based**	Mean	0.21	0.26	nd	0.68	0.28	0.22	nd	0.67	nd	0.39	0.07	0.46	0.31	0.08	0.12	nd	4.75	0.82
	SD	0.04	0.13	–	0.05	0.06	0.16	–	0.07	–	0.03	0.04	0.02	0.02	0.06	0.13	–	0.27	0.08
	Max	0.24	0.35	nd	0.71	0.32	0.33	nd	0.72	nd	0.41	0.09	0.47	0.32	0.12	0.21	nd	4.94	0.88
	Min	0.18	nd	nd	0.64	0.24	nd	nd	0.62	nd	0.37	nd	0.44	0.29	nd	nd	nd	4.56	0.76
**Mixed wheat and rice-based**	Mean	0.10	nd	nd	nd	nd	nd	nd	0.33	nd	0.28	0.08	0.34	0.18	nd	0.08	nd	3.10	0.59
	SD	0.01	–	–	–	–	–	–	0.01	–	0.16	0.06	0.18	0.09	–	0.06	–	0.56	0.31
	Max	0.11	nd	nd	nd	nd	nd	nd	0.33	nd	0.39	0.12	0.46	0.24	nd	0.12	nd	3.49	0.81
	Min	0.09	nd	nd	nd	nd	nd	nd	0.32	nd	nd	nd	nd	nd	nd	nd	nd	2.70	0.37
**Mixed wheat and honey-based**	Mean	0.20	nd	nd	nd	nd	0.20	nd	0.52	nd	0.26	nd	0.34	nd	nd	nd	0.56	3.31	0.47
	SD	0.05	–	–	–	–	0.13	–	0.15	–	0.14	–	0.18	–	–	–	–	0.65	0.14
	Max	0.23	nd	nd	nd	nd	0.29	nd	0.62	nd	0.36	nd	0.47	nd	nd	nd	nd	3.77	0.57
	Min	0.16	nd	nd	nd	nd	nd	nd	0.41	nd	nd	nd	nd	nd	nd	nd	nd	2.85	0.37
**Mixed wheat and date-based**	Mean	nd	nd	nd	nd	nd	0.19	nd	0.38	nd	0.27	nd	nd	0.26	nd	nd	nd	3.03	0.63
	SD	–	–	–	–	–	0.11	–	0.13	–	0.15	–	–	0.01	–	–	–	0.41	0.16
	Max	nd	nd	nd	nd	nd	0.27	nd	0.47	nd	0.37	nd	nd	0.27	nd	nd	nd	3.32	0.74
	Min	nd	nd	nd	nd	nd	nd	nd	0.28	nd	nd	nd	nd	0.25	nd	nd	nd	2.74	0.51
**Mixed almond porridge and fruit-based**	Mean	0.11	nd	nd	0.49	nd	0.20	nd	0.35	nd	nd	0.09	0.44	0.55	0.07	nd	nd	3.74	0.86
	SD	0.09	–	–	0.26	–	0.12	–	0.08	–	–	0.06	0.01	0.04	0.04	–	–	0.18	0.11
	Max	0.17	nd	nd	0.67	nd	0.28	nd	0.40	nd	nd	0.13	0.45	0.58	0.09	nd	nd	3.87	0.93
	Min	nd	nd	nd	nd	nd	nd	nd	0.29	nd	nd	nd	0.43	0.52	nd	nd	nd	3.61	0.78
**Mixed wheat and fruit-based**	Mean	0.21	nd	nd	nd	nd	nd	nd	0.32	nd	0.29	0.07	nd	0.32	nd	nd	0.56	3.17	0.73
	SD	0.03	–	–	–	–	–	–	0.01	–	0.18	0.04	–	0.03	–	–	–	0.28	0.24
	Max	0.23	nd	nd	nd	nd	nd	nd	0.33	nd	0.41	0.09	nd	0.34	nd	nd	nd	3.37	0.90
	Min	0.19	nd	nd	nd	nd	nd	nd	0.31	nd	nd	nd	nd	0.30	nd	nd	nd	2.97	0.56
**Mixed 5 cereal-based**	Mean	0.25	0.31	nd	0.81	0.19	0.38	nd	0.48	nd	0.40	0.18	0.56	0.36	0.09	0.06	nd	5.06	0.98
	SD	0.08	0.20	–	0.11	0.13	0.10	–	0.28	–	0.02	0.05	0.05	0.05	0.07	0.04	–	0.68	0.02
	Max	0.30	0.45	nd	0.89	0.28	0.45	nd	0.68	nd	0.41	0.21	0.59	0.39	0.14	0.09	nd	5.54	0.98
	Min	0.19	nd	nd	0.73	nd	0.31	nd	0.28	nd	0.38	0.14	0.52	0.32	nd	nd	nd	4.58	0.97
**Almond porridge-based**	Mean	0.18	nd	nd	nd	0.23	0.29	nd	0.56	nd	0.35	0.11	0.47	0.43	nd	nd	nd	4.16	0.95
	SD	0.01	–	–	–	0.19	0.01	–	0.05	–	0.02	0.03	0.01	0.01	–	–	–	0.08	0.06
	Max	0.19	nd	nd	nd	0.36	0.29	Nd	0.59	nd	0.36	0.13	0.48	0.44	nd	nd	nd	4.22	0.99
	Min	0.17	nd	nd	nd	nd	0.28	Nd	0.52	nd	0.33	0.09	0.46	0.42	nd	nd	nd	4.10	0.90
***P*** **-value**		0.96	1.00	1.00	1.00	0.96	0.27	1.00	0.27	1.00	0.96	0.27	0.27	0.27	1.00	1.00	1.00	0.27	0.27

*PAHs, Polycyclic Aromatic Hydrocarbons; nd, non-detected*.

Very few studies have been performed on PAHs in a variety of baby foods, and two of them are mentioned below.

Badibostan et al. measured four PAHs in kinds of baby food samples in Iran and reported the mean values of BaP in wheat + honey, wheat + fruit, wheat + palm, wheat + milk, and wheat + banana were 0.35 ± 0.09, 0.19 ± 0.03, 0.48 ± 0.04, nd, and 0.19 ± 0.02 μg/kg, respectively, that were somewhat similar to our findings (18). Soceanu et al. measured 14 PAHs in kinds of baby food samples in Romania and reported the mean PAHs level in vegetable-based puree, fruit-based puree, veal-based puree, white fish-based puree, cereals, and biscuits were 4.73 ± 0.33, 5.39 ± 0.28, 2.52 ± 0.22, 6.02 ± 0.4, 4.07 ± 0.37, and 6.7 ± 0.375 μg/kg, respectively, that higher than our findings (17).

### Human Health Risk Assessment

According to the EPA procedure, Monte Carlo Simulation was performed in the probabilistic hazard evaluations to reduce the uncertainties of the hazard predictions with probability assignment for each variable to elude underestimation or overestimation (30, 32, 33, 39). For this purpose, several research studies have been done on the assessment of probabilistic health risk by using Monte Carlo Simulation for PAH_S_ in infant formulae in Iran (18), arsenic in infants Dietary intake in the USA (40), PAHs in mushrooms (raw, grilled, and fried) (8), trace elements and sulfur dioxide residual in raisins (34). The rank order of the EDI index according to percentile 95% was P > fluorene (F) > benzo[k] fluoranthene (BkF) >BaP > Ch >A > acenaphthylene (Ace) > naphthalene (NA) > phenanthrene (Pa) > BbF > indeno[1,2,3– cd]pyrene (IP) > dibenzo[a,h] anthracene (DhA), as shown in Table 6.

**Table 6:** Uncertainly analysis for the daily intake (μg/kg bw/day) of PAHs in baby foods.

	**NA**	**Ace**	**F**	**Pa**	**A**	**P**	**Ch**	**BbF**	**BkF**	**BaP**	**DhA**	**IP**
5%	1.96E-8	2.47E-8	5.13E-8	1.73E-8	2.97E-8	5.54E-8	3.53E-8	9.77E-9	4.55E-8	3.59E-8	6.19E-9	6.21E-9
50%	2.91E-8	3.69E-8	7.72E-8	2.53E-8	4.22E-8	8.37E-8	5.33E-8	1.51E-8	6.72E-8	5.47E-8	9.32E-9	9.20E-9
75%	3.45E-8	4.41E-8	9.17E-8	3.00E-8	4.94E-8	9.80E-8	6.31E-8	1.77E-8	7.78E-8	6.38E-8	1.09E-8	1.09E-8
95%	4.34E-8	5.53E-8	1.19E-7	3.75E-8	6.22E-8	1.26E-7	7.79E-8	2.20E-8	9.91E-8	8.11E-8	1.41E-8	1.43E-8

*PAHs, Polycyclic Aromatic Hydrocarbons*.

**Figure 1:**
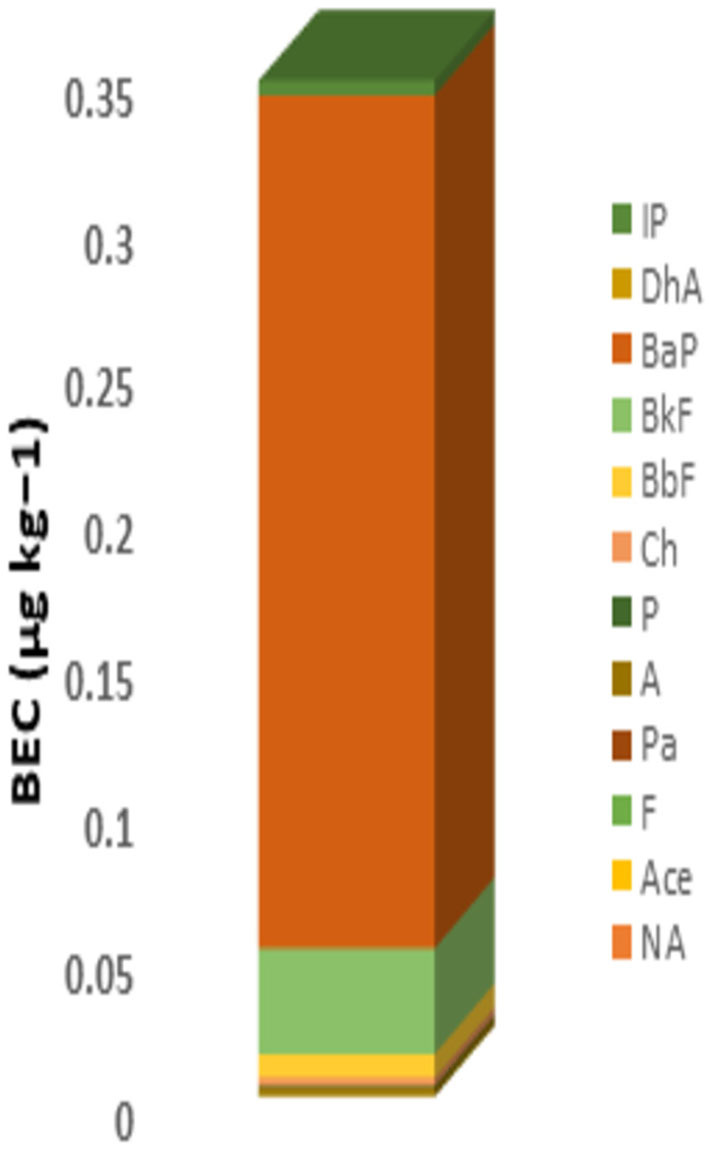
BEC value and contribution due to content of PAHs in baby food.

**Figure 2:**
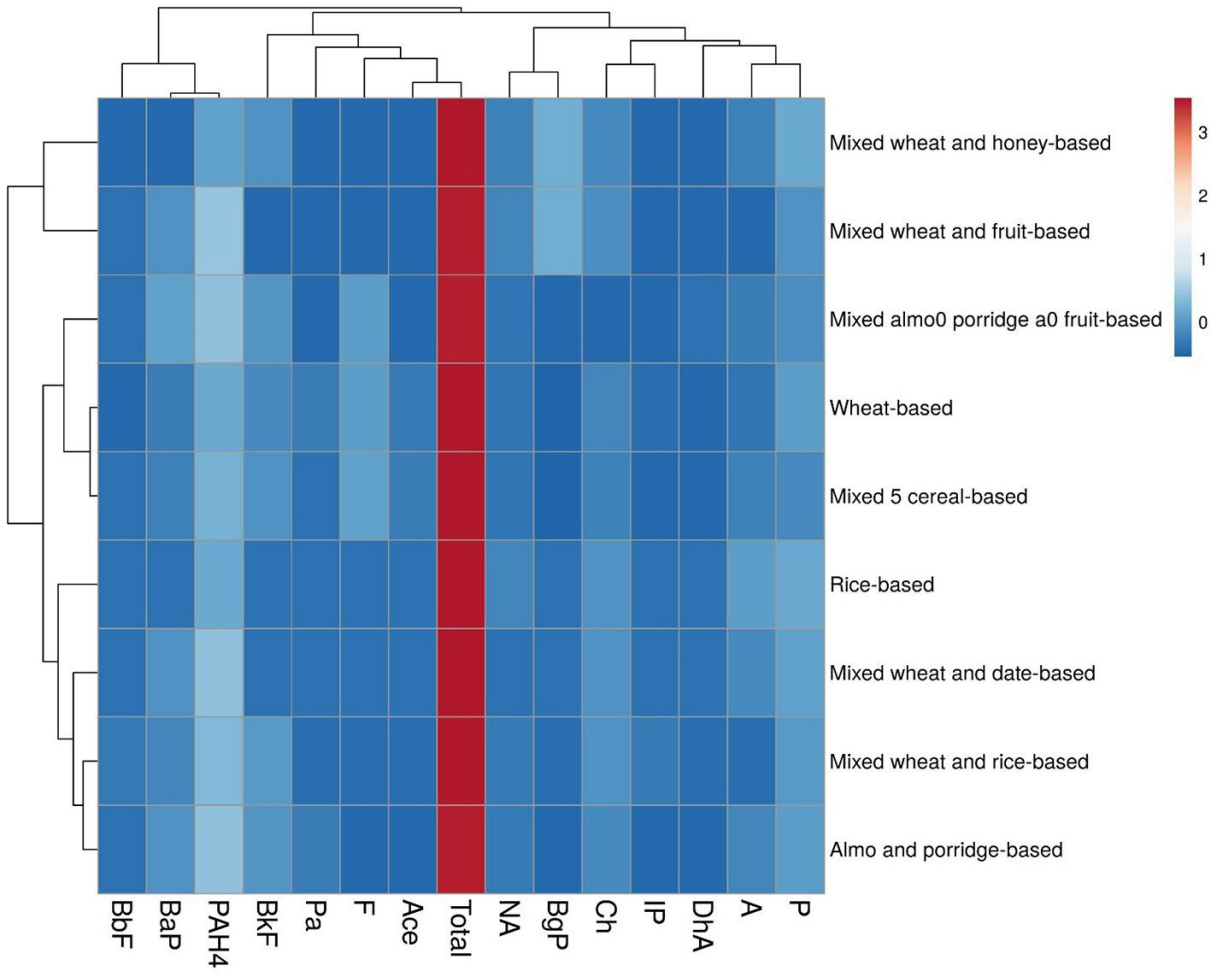
Estimation of the ILCR of PAH in baby foods.

According to the suggested Joint FAO expert Food Additives Committee, a daily dietary exposure to BaP over 10 ng/kg bw/day is required to begin dangers to human health. Among all samples, EDI values were below 1; consequently, baby food was not dangerous due to PAHs to the public's health. Figure 1 shows that the BaP and BkF 7 are the two principal contributors to the total BEC (μg/kg), while the other PAHs included have a contribution of lower than 5%. Based on the simulation of Monte Carlo results, the indexes of ILCR (percentile 95%) in the baby food was 7.03E-10. The probabilistic distributions and simulation histogram of BaP risk for the baby are shown in Figure 2. The qualitative classification of carcinogenic risk terms can describe in three forms; the ILCR indexes with a value lower than 10^−6^ is the safe zone; the ILCR indexes with a value higher than 10^−4^ is the limit of threshold risk; the ILCR indexes higher than 10^−3^ is the zone of significant danger. In similar studies in Italy, Santonicola showed that carcinogenic risk due to PAHs in milk and baby food samples based on meat/fish was a potential concern for baby health (22). In China, Cai et al. (19) showed that the risk assessment for dietary exposure to contamination of PAHs due to the ingestion of foreign and domestic powders of infant formula presented low-risk levels for consumption (19).

### Multivariate Analysis

**Figure 3:**
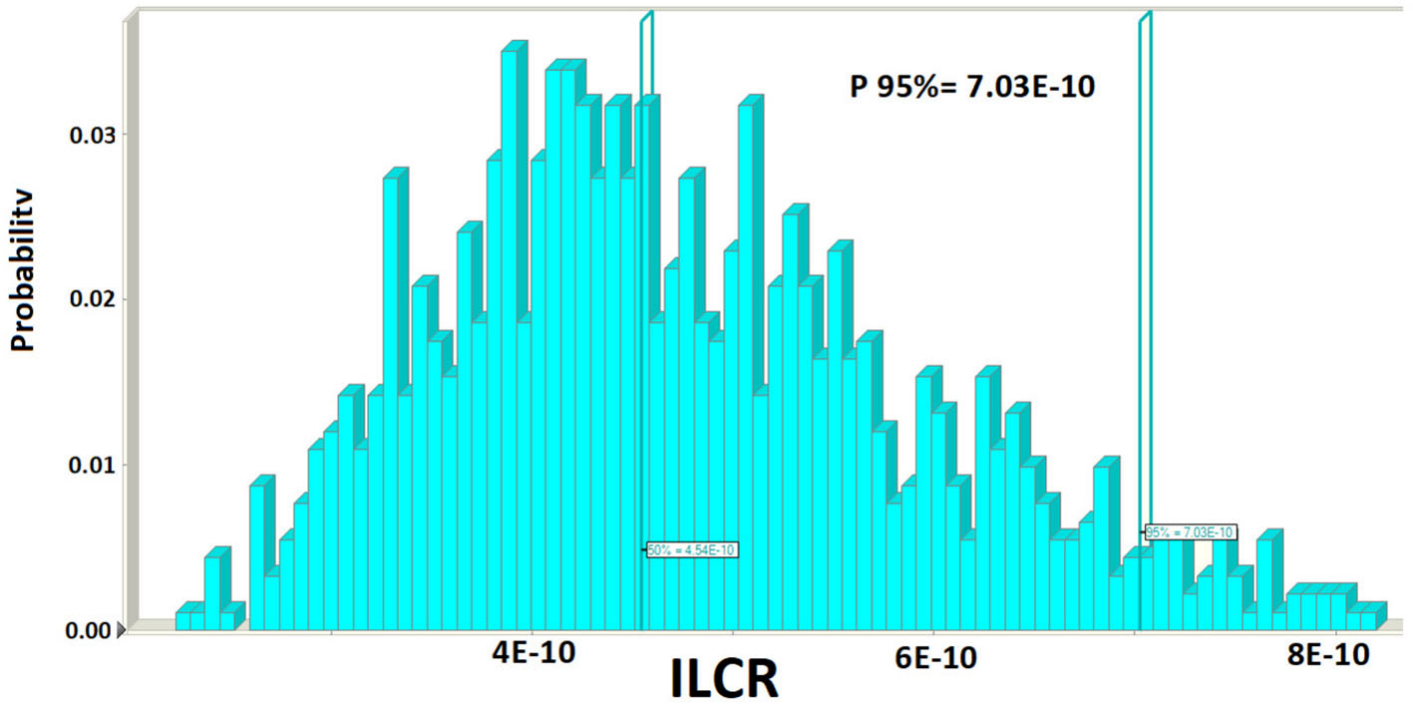
Heat map of PAH in baby foods.

The heat map was applied to understand the PAH compound's associations in various baby food samples. A heat map visualization, in addition to classification rows and columns of similar parameters, gives a comprehensive pattern of the highest and least variables in the generating model. Moreover, heat maps explained the food baby samples (rice-based, wheat-based, mixed wheat and rice-based, mixed wheat and honey-based, mixed wheat and date-based, mixed almond porridge and fruit-based, mixed wheat and fruit-based, mixed five cereal-based, and almond porridge-based) were independent variables in the PAH congener clustering. The heat map collected samples of baby foods into three major clusters (Figure 3). The first cluster includes BbF, BaP, and PAH4, second cluster includes BkF, Pa, Ace, F, and total PAH, and the third cluster includes NA, DhA, IP, BgP, P, and Ch. Clust Vis was employed to visualize the clustering of related data. The BbF, BaP, and PAH4 groups were the closers, showing that the frequency variations of these PAH compounds had a similar trend in various samples.

## Conclusions

The present study for the first time compared the levels of PAHs (16 compounds) in baby food samples collected in the Iranian market. To extract 16 PAH compounds, the MSPE-GC/MS approach was devised that gave a clean extraction and better than 93.4% recoveries for PAH analytes. In all samples, BaP was lower than the EU standard limit (1 μg/kg). Moreover, the results showed that there was no significant difference between the different baby food samples, and in all samples, the level of PAHs was lower than the existing standards. Furthermore, the situation of PAH congeners on the heat map showed special trends in analogous parameters and visualized the research items among groups. The health risk results based on the Monte Carlo simulation were exhibited in which the ILCR value was lower than 10^−6^. Therefore, consuming the examined baby food samples was not a significant carcinogenic risk for consumers. Despite the results of this study, it is recommended that due to the high risks of these contaminants to humans especially babies and infants, further research and monitoring of baby food and milk and milk powdered throughout Iran.

## Data Availability Statement

The raw data supporting the conclusions of this article will be made available by the authors, without undue reservation.

## Author Contributions

MM: conceptualization, methodology and software, writing—original draft preparation, and data curation. MAr: investigation and visualization. NS: design of study, supervision, and re-writing—original draft preparation. HH: software and validation. MAh: writing—original draft preparation. All authors contributed to the article and approved the submitted version.

## Funding

This study has been supported by the Tehran University of Medical Sciences (TUMS).

## Conflict of Interest

The authors declare that the research was conducted in the absence of any commercial or financial relationships that could be construed as a potential conflict of interest.

## Publisher's Note

All claims expressed in this article are solely those of the authors and do not necessarily represent those of their affiliated organizations, or those of the publisher, the editors and the reviewers. Any product that may be evaluated in this article, or claim that may be made by its manufacturer, is not guaranteed or endorsed by the publisher.
