# Supplementary material for: Measurement of Polycyclic Aromatic Hydrocarbons in Baby Food Samples in Tehran, Iran With Magnetic-Solid-Phase-Extraction and Gas-Chromatography/Mass-Spectrometry Method: A Health Risk Assessment
Source: Front Nutr. 2022 Feb 17;9:833158. doi: 10.3389/fnut.2022.833158 (PMC8891379; doi:10.3389/fnut.2022.833158)
Supplement: Supplementary file 1 [file Table_1.docx]

**Table S1: PAHs and their toxic equivalent factors (TEFs)**

| PAHs | TEF | PAHs | TEF |
| --- | --- | --- | --- |
| Benzo(a)pyrene (BaP) | 1 | Anthracene (A) | 0.01 |
| Dibenz(a,h)anthracene (DahA) | 1 | Naphthalene (NA) | 0.001 |
| Benzo(k)fluoranthene (BkF) | 0.1 | Acenaphthylene (AC) | 0.001 |
| Indeno(l,2,3-cd)pyrene (IcdP) | 0.1 | Acenaphthene (ACE) | 0.001 |
| Benz(a)anthracene (BaA) | 0.1 | Phenanthrene (PHE) | 0.001 |
| Benzo(b)fluoranthene (BbF) | 0.1 | Fluorine (FLO) | 0.001 |
| Chrysene (CHR) | 0.01 | Pyrene (PYR) | 0.001 |
| Benzo(g,h,i)perylene (BghiP) | 0.01 | Fluoranthene (FL) | 0.001 |
